# Extracts of *Crataegus oxyacantha* and *Rosmarinus officinalis* Attenuate Ischemic Myocardial Damage by Decreasing Oxidative Stress and Regulating the Production of Cardiac Vasoactive Agents

**DOI:** 10.3390/ijms18112412

**Published:** 2017-11-14

**Authors:** Raúl Enrique Cuevas-Durán, Juan Carlos Medrano-Rodríguez, María Sánchez-Aguilar, Elizabeth Soria-Castro, María Esther Rubio-Ruíz, Leonardo Del Valle-Mondragón, Alicia Sánchez-Mendoza, Juan Carlos Torres-Narvaéz, Gustavo Pastelín-Hernández, Luz Ibarra-Lara

**Affiliations:** 1 Unidad Académica de Medicina Humana y Ciencias de la Salud, Universidad Autónoma de Zacatecas, Zacatecas 98000, Mexico; raul.e.cd@hotmail.com (R.E.C.-D.); merodi12@hotmail.com (J.C.M.-R.); 2 Department of Pharmacology, Instituto Nacional de Cardiología Ignacio Chávez, Juan Badiano 1, Sección XVI, Tlalpan, Mexico City 14080, Mexico; msanchezaguilar@gmail.com (M.S.-A.); leonardodvm65@hotmail.com (L.D.V.-M.); masanchez@gmail.com (A.S.-M.); juancarlostn63@hotmail.com (J.C.T.-N); pastelingustavo@gmail.com (G.P.-H.); 3 Department of Pathology, Instituto Nacional de Cardiología Ignacio Chávez, Juan Badiano 1, Sección XVI, Tlalpan, Mexico City 14080, Mexico; sorieli@hotmail.com; 4 Department of Physiology, Instituto Nacional de Cardiología Ignacio Chávez, Juan Badiano 1, Sección XVI, Tlalpan, Mexico City 14080, Mexico; esther_rubio_ruiz@yahoo.com

**Keywords:** *Crataegus oxyacantha*, *Rosmarinus officinalis*, myocardial function, antioxidant status, vasoconstrictor, vasodilators

## Abstract

Numerous studies have supported a role for oxidative stress in the development of ischemic damage and endothelial dysfunction. *Crataegus oxyacantha* (*Co*) and *Rosmarinus officinalis* (*Ro*) extracts are polyphenolic-rich compounds that have proven to be efficient in the treatment of cardiovascular diseases. We studied the effect of extracts from *Co* and *Ro* on the myocardial damage associated with the oxidative status and to the production of different vasoactive agents. Rats were assigned to the following groups: (a) sham; (b) vehicle-treated myocardial infarction (MI) (MI-V); (c) *Ro* extract-treated myocardial infarction (MI-*Ro*); (d) *Co* extract-treated myocardial infarction (MI-*Co*); or (e) *Ro+Co*-treated myocardial infarction (MI-*Ro+Co*). *Ro* and *Co* treatments increased total antioxidant capacity, the expression of superoxide dismutase (SOD)-Cu^2+^/Zn^2+^, SOD-Mn^2+^, and catalase, with the subsequent decline of malondialdehyde and 8-hydroxy-2′-deoxyguanosine levels. The extracts diminished vasoconstrictor peptide levels (angiotensin II and endothelin-1), increased vasodilators agents (angiotensin 1–7 and bradikinin) and improved nitric oxide metabolism. Polyphenol treatment restored the left intraventricular pressure and cardiac mechanical work. We conclude that *Ro* and *Co* treatment attenuate morphological and functional ischemic-related changes by both an oxidant load reduction and improvement of the balance between vasoconstrictors and vasodilators.

## 1. Introduction

Myocardial infarction is the leading cause of death in developed nations. This event results from the abrupt reduction of coronary flow in a segment of the myocardium. Consequently, severe cellular changes ensue and inevitably end in cell death and necrosis [1]. The damage that occurs in the myocardium due to ischemia is mainly due to an increase in the levels of reactive oxygen species (ROS) [2]. ROS can be produced in the cell by various enzymes including NADPH oxidase (NOX) and nitric oxide synthase (NOS) owing to a lack of tetrahydrobiopterin cofactor (BH_4_) [3,4,5]. Indeed, ROS induce a state of oxidation that can lead to cellular membrane injury with the consequent alteration in metabolic processes.

Several reports suggest that an antioxidant therapy prior to ischemia may help the myocardium to recover from the damage produced by ROS [6]. In fact, deleterious effects of ROS on cardiac tissue can be blocked by antioxidant enzymes, such as superoxide dismutase (SOD) and catalase [7].

Both endothelin-1 (ET-1) and angiotensin II (Ang II) have been found to be potent vasoconstrictors, whose concentrations increase in the heart under ischemic conditions [8,9]. There is also evidence that Ang II induces oxidative stress during ischemia by the activation of NOX via Ang II type-1 receptor (AT1) [10]. In consequence, this generates an Ang II-induced myocyte edema, which damages the gap junction, the impulse of conduction, and the activation of ion channels. These effects promote an increase of cardiac arrhythmias during ischemia. On the other hand, the angiotensin converting enzyme (ACE)—producer of Ang II—deactivates bradykinin production; bradykinin is a peptide that may induce tissue protection during ischemia by promoting the release of prostaglandins which, in turn, improve blood flow and oxygen delivery to the heart [11]. Conversely, it has been shown that angiotensin-(1–7) counteracts the effects of Ang II [12], reduces cell volume and decreases the activation of ion channels, possibly decreasing cardiac arrhythmias during ischemia.

Plant extracts are known to exhibit a wide array of pharmacological effects like hypolipidemic, antioxidant, antiproliferative, and anti-inflammatory. The leaves, berries, and flowers of *Crataegus oxycanta* (*Co*) have been used traditionally in a variety of functional heart disorders. Flavonoids and procyanidins have been proposed to be the most active constituents of *Co* [13,14]. Several studies have shown that *Co* extract is effective in quenching ROS [15,16]. Moreover, Elango et al. [17] have shown that the alcoholic extract of *Co* is able to protect the brain from ischemia reperfusion injury in a rat stroke model.

Extracts of *Rosmarinus officinalis* (*Ro*) have been widely used as a preservative in the food industry because of the antioxidant activity of some of its components, for instance, diterpenes carnosol and carnosic acid [18,19]. These extracts also consist of rosmarinic acid, which is structurally the ester of the antioxidants caffeic acid and 3,4-dihydroxyphenyl-lactic acid. In in vitro oxidative stress models, rosmarinic acid has been used as a good scavenger of peroxyl radicals and has shown its ability to block the formation of hydroxyl radicals [18,20,21].

On the whole, there is a need to understand and identify suitable antioxidant interventions to salvage the myocardium from ischemia damage and dysfunction. Therefore, this work focuses on ascertaining the effect of extracts of *Co* and *Ro* on the myocardial damage induced by ischemia associated with an oxidative status and to the production of different vasoactive agents.

## 2. Results

### 2.1. Qualitative and Quantitative Analysis of the Extracts from Ro and Co

**Figure 1:**
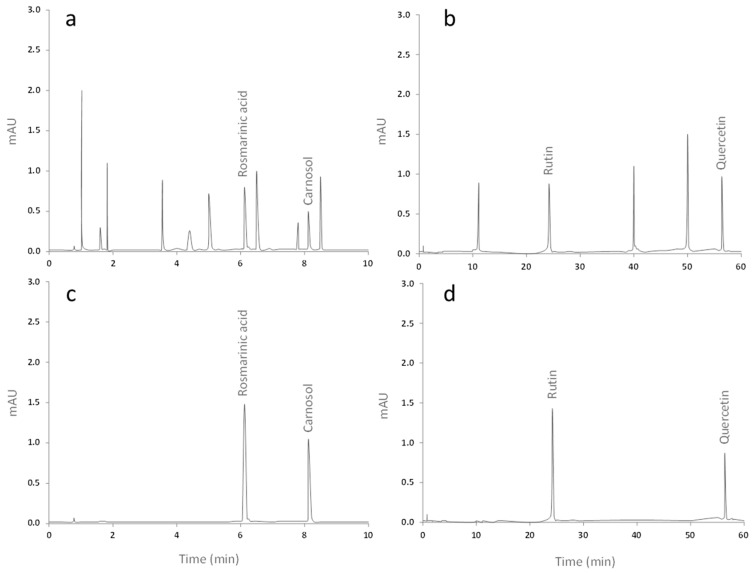
Chromatography of the foliar extract of *Rosmarinus officinalis* (**a**) compared to the chromatogram at 280 nm of the standards rosmarinic acid and carnosol (**c**); *Crataegus oxyacantha* (**b**) compared to the chromatogram at 360 nm of the rutin and quercetin standards (**d**).

High-performance liquid chromatography (HPLC) analysis showed that *Ro* extract contains rosmarinic acid and carnasol, while *Co* extract exhibits the presence of rutin and quercetin (Table 1). The chemical identification of the compounds was achieved comparing the retention times of reference standards to those contained in the extracts (Figure 1d).

### 2.2. Effect of Ro, Co, and Their Combination in Myocardial Oxidative Stress

**Figure 2:**
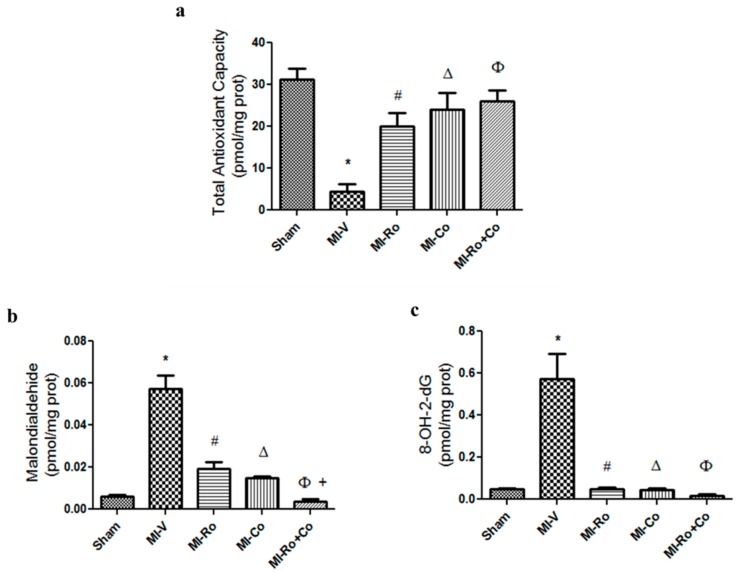
The effect of extracts from *Rosmarinus officinalis*, *Crataegus oxyacantha,* and their combination on oxidative stress markers. Total antioxidant capacity (TAC) (**a**); malondialdehyde (MDA) (**b**); and 8-hydroxy-2′-deoxyguanosine (8-OH-2dG) (**c**); The concentrations were evaluated in the ischemic left ventricle areas from rats subjected to sham—or myocardial infarction (MI) and treated seven days with either vehicle (V), *Rosmarinus officinalis* (*Ro*), *Crataegus oxyacantha* (*Co*) or their combination (*Ro+Co*). The values show the mean ± SEM from 6 different experiments. *p* < 0.05 ANOVA one way post Tukey, * Sham vs. MI-V, # MI-V vs. MI-*Ro*, Δ MI-V vs. MI-*Co*, Φ MI-V vs. MI-*Ro+Co, +* MI*-Ro* vs. MI*-Ro+Co.*

To assess myocardial oxidative stress in the homogenates of ventricles of all experimental groups, we determined total antioxidant capacity (TAC), malondialdehyde (MDA), and 8-hydroxy-2′-deoxyguanosine (8-HO-2dG) levels (Figure 2a–c, respectively). As expected, MI lowered TAC; this event was prevented with *Ro*, *Co,* and *Ro+Co* treatment. Ischemia significantly increased MDA and 8HO-2dG concentrations compared to sham animals. *Ro* and *Co* treatment significantly decreased these parameters; however, *Ro+Co* had a synergistic effect by further decreasing the levels of MDA when compared to the natural compounds alone.

**Figure 3:**
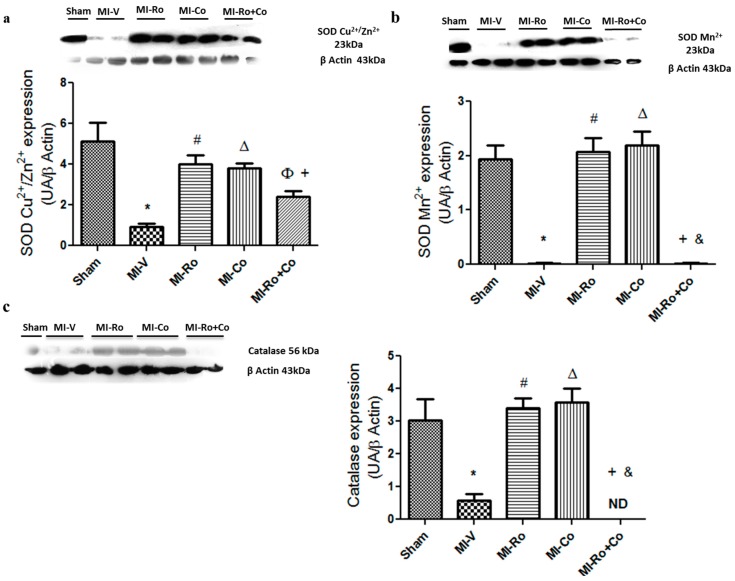
The effect of extracts from *Rosmarinus officinalis* (*Ro*), *Crataegus oxyacantha* (*Co*), and their combination (*Ro+Co*) on superoxide dismutase (SOD)-Cu^2+^/Zn^2+^ (**a**); SOD-Mn^2+^ (**b**); and catalase (**c**) protein expression in ischemic left ventricle area from sham, myocardial infarction (MI)-vehicle treated, MI-*Ro*, MI-*Co*, and MI-*Ro+Co* experimental groups. The values show the mean ± SEM (*n* = 6 per group). *p* < 0.05 ANOVA one way post Tukey * Sham vs. MI-V, # MI-V vs. MI-*Ro*, Δ MI-V vs. MI-*Co*, Φ MI-V vs. MI-*Ro+Co*, + MI-*Ro* vs. MI-*Ro+Co*, & MI-*Co* vs. MI-*Ro+Co*. ND (Not detected).

Additionally, we analyzed the expression of myocardial SOD-Cu^2+^/Zn^2+^, SOD-Mn^2+^, and catalase. Figure 3a–c show that the expression of these enzymes was significantly diminished in the MI group when compared to the sham group. The administration of *Ro* and *Co*, was able to prevent a decline in the expression of the three antioxidant enzymes, maintaning values similar to those of the sham group. Interestingly, a combination of the compounds (*Ro+Co*) was not capable of producing the same effect that polyphenols alone; futhermore, our results show that there was no catalase expression in MI-*Ro+Co* group (Figure 3c).

### 2.3. Effect of Natural Compound Administration on Vasoconstrictor Agents Production

**Figure 4:**
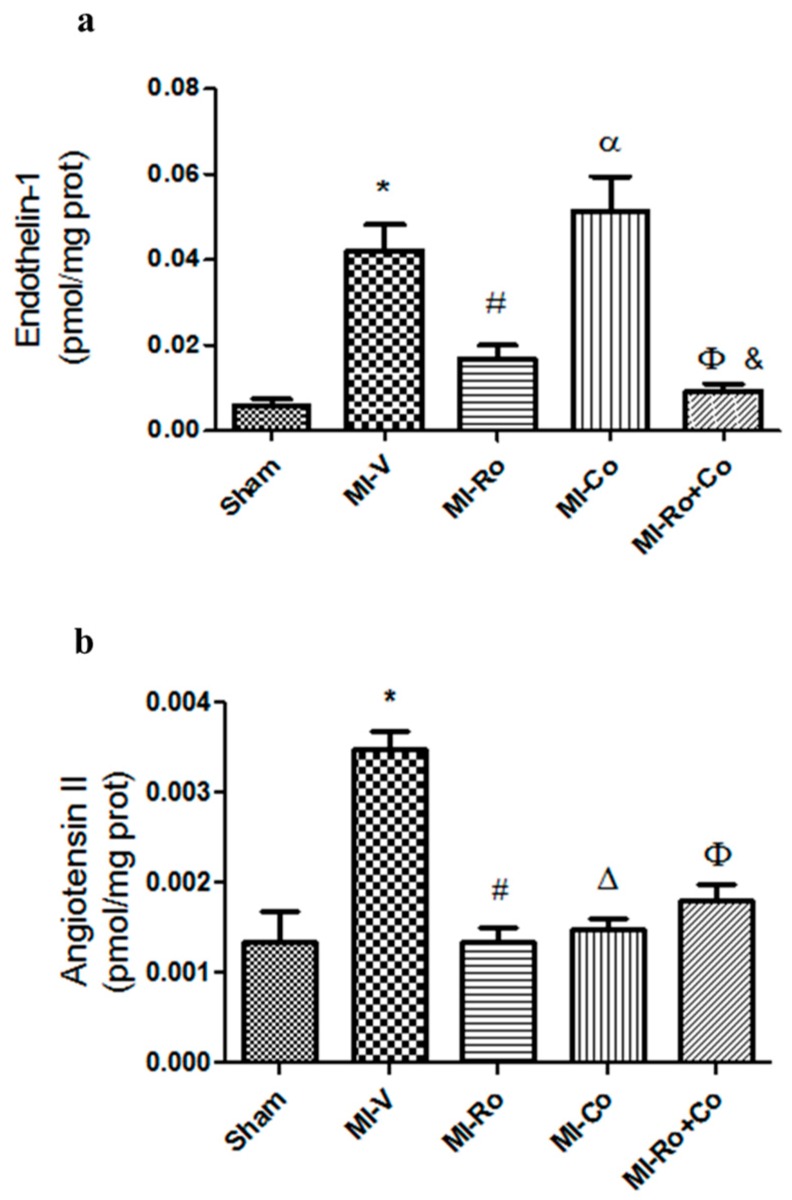
The effect of extracts from *Rosmarinus officinalis* (*Ro*), *Crataegus oxyacantha* (*Co*) and their combination (*Ro+Co*) on vasoconstrictor agent levels. Endothelin-1 (**a**) and angiotensin II (**b**) concentration were determined in myocardial ischemic area homogenate from sham, myocardial infarction (MI)-vehicle treated, MI-*Ro*, MI-*Co*, and MI-*Ro+Co* experimental group. The values show the mean ± SEM from six different experiments. *p* < 0.05 ANOVA one way post Tukey, * Sham vs. MI-V, # MI-V vs. MI-*Ro*, Δ MI-V vs. MI-*Co*, Φ MI-V vs. MI-*Ro*+*Co*, α MI-*Ro* vs. MI-*Co*, & MI-*Co* vs. MI-*Ro+Co*.

Figure 4 shows Ang II and ET-1 concentrations in every experimental group. The data show that vasoconstrictor agent production increased in the left ventricles from rats under ischemic conditions. *Ro* and *Ro+Co* significantly diminished ET-1 concentration; however, *Co*-treatment augmented ET-1 production in comparison to sham rats (Figure 4a). With respect to Ang II production, treatment with extracts restored the levels of this peptide in the left ventricles of treated rats (Figure 4b).

**Figure 5:**
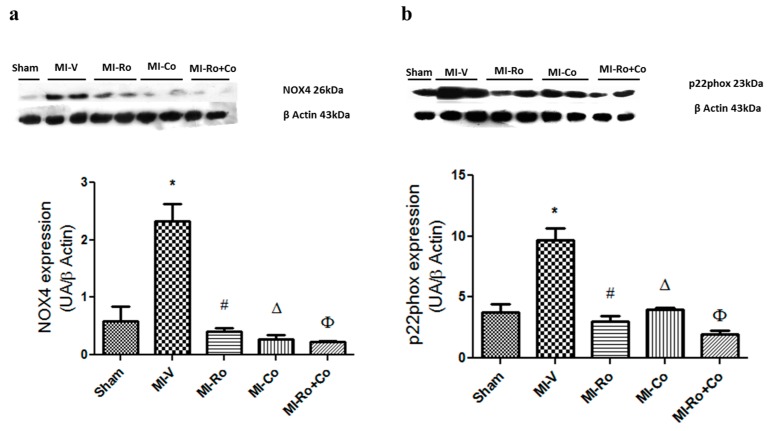
The effect of extracts from *Rosmarinus officinalis* (*Ro*), *Crataegus oxyacantha* (*Co*), and their combination (*Ro+Co*) on NADPH oxidase (NOX4) (**a**) and p22phox (**b**) protein expression in ischemic area of the left ventricles from sham, myocardial infarction (MI)-vehicle treated, MI-*Ro*, MI-*Co*, and MI-*Ro+Co* experimental groups. The values show the mean ± SEM of six different experiments. *p* < 0.05 ANOVA one way post Tukey, * Sham vs. MI-V, # MI-V vs. MI-*Ro*, Δ MI-V vs. MI-*Co*, Φ MI-V vs. MI-*Ro+Co*.

Next, we investigated whether Ang II concentrations could be associated with the expression of NADPH oxidase subunits (NOX) 4 and (p22phox), subunits which are activated by the peptide. Our results showed a significant increase in the expression of NOX4 and p22phox in the MI-V group when compared to the sham group; the administration of either compound significantly diminished the expression of both proteins (Figure 5a,b).

### 2.4. Effect of Natural Compound Administration on Ang-(1–7), Bradykinin, and the eNOS Pathway

**Figure 6:**
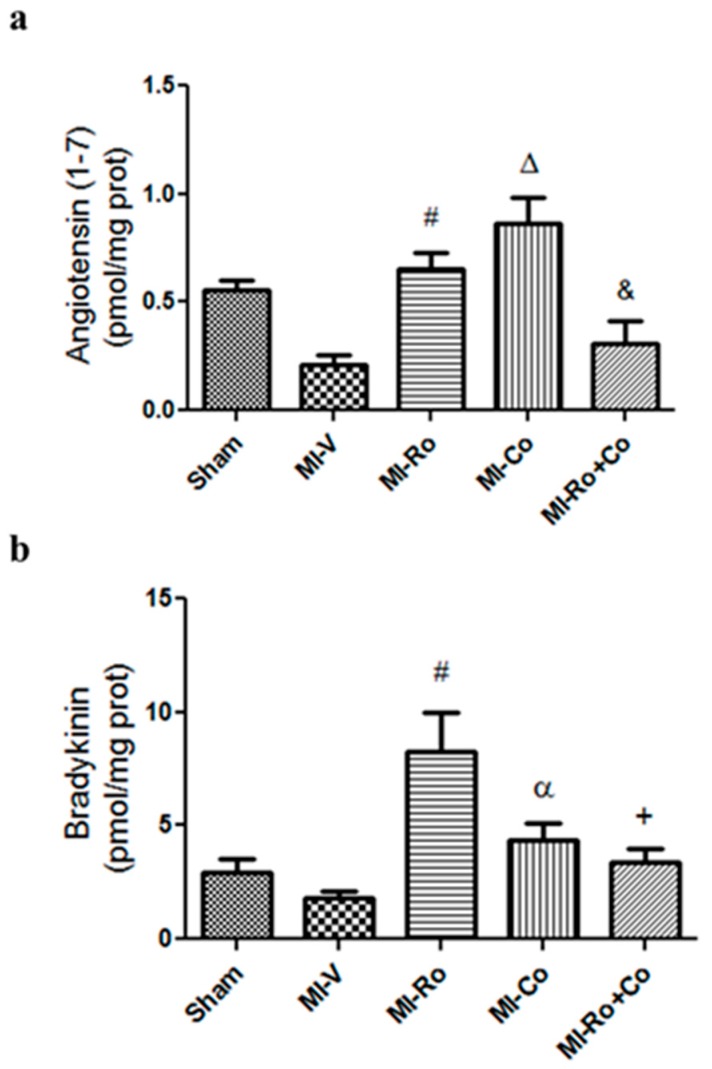
The effect of extarcts from *Rosmarinus officinalis* (*Ro*), *Crataegus oxyacantha* (*Co*), and their combination (*Ro+Co*) on vasorelaxing components concentration. Angiotensin-(1–7) (**a**) and bradykinin (**b**) were quantified in the myocardial ischemic areas from sham, myocardial infarction (MI)-vehicle treated (MI-V), MI-*Ro*, MI-*Co*, and MI-*Ro+Co*. The values correspond to the mean ± SEM of six different experiments. *p* < 0.05 ANOVA one way post Tukey # MI-V vs. MI-*Ro*, Δ MI-V vs. MI-*Co*, + MI-*Ro* vs. MI-*Ro+Co*, α MI-*Ro* vs. MI-*Co*, & MI-*Co* vs. MI-*Ro+Co*.

We evaluated the effect of *Ro*, *Co*, and *Ro+Co* administration on vasodilator mediators. When the hearts were subjected to ischemia, we observed a clear tendency towards diminished values of Ang-(1–7) and bradykinin (Figure 6a,b, respectively). Extracts of *Ro* and *Co* significantly increased Ang-(1–7) concentration; meanwhile *Ro+Co* administration had no effect on this parameter (Figure 6a). Figure 6b shows that only *Ro* extract significantly augmented bradykinin concentration.

**Figure 7:**
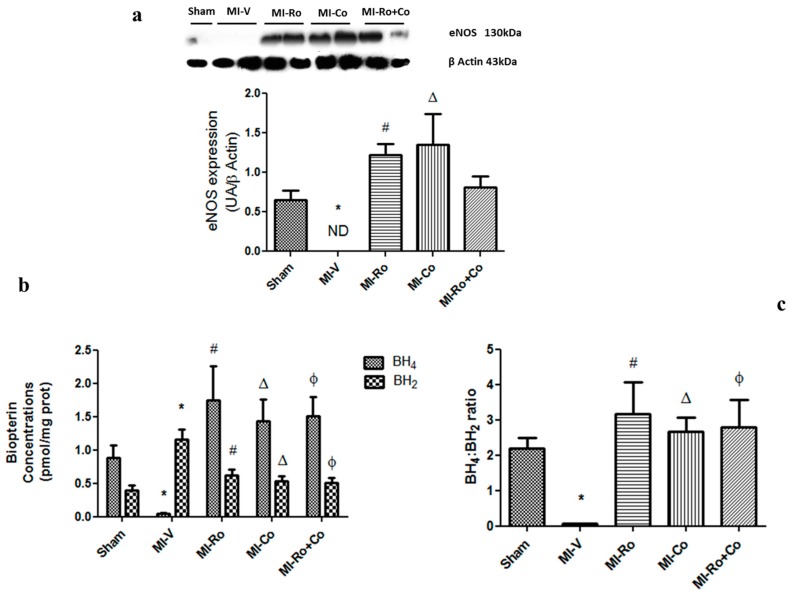
Extracts of *Rosmarinus officinalis* (*Ro*), *Crataegus oxyacantha* (*Co*), and their combination (*Ro+Co*) increases endothelial nitric oxide synthase (eNOS) expression and BH_4_:BH_2_ ratio. (**a**) Representative immunoblot and eNOS expression; (**b**) biopterin concentrations; and (**c**) BH_4_:BH_2_ ratio in ischemic areas from sham, myocardial infarction (MI)-vehicle (MI-V)-treated, MI-*Ro*, MI-*Co*, and MI-*Ro+Co*. The values correspond to the mean ± SEM of six rats/group. *p* < 0.05 ANOVA one way post Tukey, * Sham vs. MI-V, # MI-V vs. MI-*Ro*, Δ MI-V vs. MI-*Co*, Φ MI-V vs. MI-*Ro+Co*.

Ischemia inhibited endothelial nitric oxide synthase (eNOS) expression in hearts from the MI-V group, whereas treatment with natural compounds improved the expression of eNOS, bringing values closer to those of controls (Figure 7a). It is known that in order to produce NO, eNOS requires the presence of BH_4_. In our study, MI affected the production of BH_4_ and BH_2_ in an opposite way. While BH_4_ decreased with the episode of ischemia, BH_2_ augmented (Figure 7b). Both of these effects were abolished by the treatment with *Ro*, *Co*, and *Ro+Co* treatments (Figure 7b,c).

### 2.5. Ultraestructural Analysis

**Figure 8:**
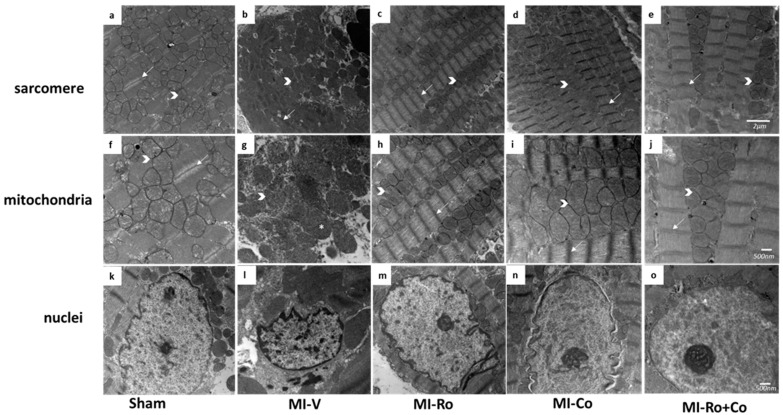
Extracts prevent ultrastructural damage in myocardium of infarcted rats. Sham (**a**,**f**,**k**); myocardial infarction (MI)-vehicle treated (MI-V, (**b**,**g**,**l**)); MI-*Ro* (**c**,**h**,**m**); MI-*Co* (**d**,**i**,**n**); and MI-*Ro+Co* (**e**,**j**,**o**) were analyzed by electron microscopy. The figure depicts the sarcomere showing mitochondria arranged in rows (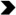); band I (→) and * showed swealling mitochondria. Magnification 12,000× for sarcomere (**a**–**e**), and the scale bar represents 500 nm; 20,000× for mitochondria and nuclei, and the scale bar represents 2 µm (**f**–**o**).

In all of the groups, the ischemic myocardial area was analyzed by electron microscopy. We observed that the sham group maintained the alignment of structure of the sarcomere and mitochondria (Figure 8a,f). Figure 8b,g,l show that the MI-V group had a generalized edema, loss of the structure of the sarcomere, shortened fibers and, therefore, increased sarcoplasmic space. These figures also illustrate swelling of mitochondria; in addition, chromatin underwent condensation in the periphery of the nuclear membrane. The group MI-*Ro* shows a structure similar to the sham group with a shortening of the sarcomere and loss of band I (Figure 8c,h,m); in the group MI-*Co*, mitochondria were slightly swollen and lost density (Figure 8d,i,n). Finally, the MI-*Ro+Co* group was similar to the sham group (Figure 8e,j,o).

### 2.6. Effect of Ro, Co, and Their Combination (Ro+Co) on Cardiac Function

To ascertain whether natural extracts induced biochemical and morphological changes which exert an influence on cardiac physiological parameters, we evaluated cardiac functioning ex vivo. Table 2 shows that Left ventricular pressure (LVP) and cardiac mechanical work (CMW) decreased significantly in hearts under ischemic conditions compared to the sham-group (56.8 ± 3.8 vs. 74.0 ± 1.3 and 19,085.0 ± 1281.2 vs. 24,808 ± 482.4 mmHg, respectively). The treatment with *Ro*, *Co*, and their combination tended to normalize these parameters. Meanwhile, CPP and CVR slightly increased during ischemia compared with sham group (88.0 ± 4.6 vs. 66.6 ± 5.3 and 8.8 ± 0.5 vs. 6.7 ± 0.5 mmHg, respectively). However, the treatment with natural compounds had no significant effect on these parameters.

## 3. Discussion

In the present work, we demonstrated that foliar extracts of *Crataegus oxycantha*, *Rosmarinus officinalis*, and their combination favor both an antioxidant environment that increases the production of vasodilators, such as Ang-(1–7) and bradykinin, and decrease the concentration of potent vasoconstrictors, such as Ang II and ET-1. These effects contribute to the protection observed in the cardiac ultrastructure, as well as to the improvement in the hemodynamic parameters studied.

### 3.1. Effect of Ro, Co, and Their Combination in Myocardial Oxidative Stress

Myocardial ischemia describes a condition in which oxygen in the heart is insufficient to maintain cellular oxidation. Alterations that occur during myocardial ischemia facilitate the formation of ROS [22]. Particularly, Zweier et al. [7] have reported that after 10 min of myocardial ischemia, concentrations of ROS increase three times their basal value, causing damage to the myocardium. Our study demonstrated that in the infarcted rats, the antioxidant capacity decreased and the production of myocardial MDA augmented, which is directly related to a rise in the formation of peroxidized lipids. We also observed ROS damage to the DNA due to an increase in the production of 8-OH-2dG. Taken together, these factors contributed to massive damage in important macromolecules and, consequently, to a decrease in antioxidant defense (Figure 2a–c).

Our results support those obtained by Swaminathan et al. [23], who reported that the ethanol extract of *Co* protected the ischemia/reperfusion heart from damage caused by ROS. Notably, recent studies in our laboratory [24] showed that, in a model of cirrhosis produced by carbon tetrachloride (CCl_4_), the treatment with an ethanolic extract of *Ro* both decreased lipid peroxidation in liver and histological changes, and normalized altered biochemical parameters.

To reduce oxidative stress, the presence of antioxidant enzymes, such as SOD, catalase and glutathione peroxidase is necessary. Recent evidence obtained using genetically-engineered animal models demonstrates the importance of catalase and SOD in protecting the myocardium against ischemia damage [25]. In fact, it has been shown that both the SOD protein and its gene expression are attenuated in the infarcted myocardium [26,27]. Furthermore, Wang et al. [27] reported that overexpression of SOD-Cu^2+^/Zn^2+^ avoids ischemia tissue damage; it was also described that this enzyme selectively reacts with anion superoxide and prolongs the half-life of NO [28]. Alternatively, SOD-Mn^2+^ is a mitochondrial antioxidant enzyme essential for maintenance and normal cellular functioning. In fact, there is evidence that shows that overexpression of the SOD-Mn^2+^ gene is beneficial in several animal models with heart disease [29]. Catalase also plays a very important role in the protection of the heart in ischemia-reperfusion processes [30].

Our results demonstrate that while the ischemia process decreases the expression of SOD-Cu^2+^/Zn^2+^, SOD-Mn^2+^ and catalase, the expression of SOD-Cu^2+^/Zn^2+^ increases significantly as a result of the treatments with foliar extracts. On the other hand, SOD-Mn^2+^ and catalase only augment their expression in those subjects treated with individual foliar extracts, whereas the combination of these compounds inhibited the expression of all these antioxidant enzymes (Figure 3a–c). Regarding this result, we suggest that since the combination of the foliar extracts diminished the expression of SOD-Cu^2+/^Zn^2+^ and SOD-Mn^2+^, then the generation of H_2_O_2_ decreased, the expression of catalase should also be reduced, even at levels that we could not detect by Western blot analysis (Figure 3c). Even though SOD-Mn^2+^ has been related to an antioxidant function, [31,32] the overexpression of SOD-Mn^2+^ in the mitochondria could lead to exacerbated oxidative damage, as well as peroxidase activity. This observation is in clear contrast to numerous studies supporting the hypothesis that SOD-Mn^2+^ is, per se, an antioxidant. Since then, the idea that SOD-Mn^2+^ can display a pro-oxidant peroxidase activity has been confirmed by other groups.

### 3.2. Effect of Natural Compound Administration on Vasoconstrictor Agents

In this work, we also evaluated the concentration of vasoactive peptides, such as Ang II and ET-1, which are both related to vasoconstriction. It has been shown that the concentration of ET-1 increases 3–4 h after the ischemic event, reaching a maximum in the first 24 h and remaining high after 48 h [8]. Our results demonstrate that this vasoconstrictor peptide increases significantly in infarcted animals, and that treatment with *Ro*, *Co*, and their combination significantly decrease its concentration (Figure 4a). Interestingly, this result is similar to that obtained by Corder et al. [33]. In their work, they evaluated the inhibitory effects of *Co* in an endothelial cell culture and demonstrated that *Co* is a potent inhibitor of ET-1 synthesis. Furthermore, Alici et al. [34] determined the effects of caffeic acid phenethyl ester (CAPE), one of the main components of *Ro*, on the concentration of ET-1 in a rat model. They concluded that the treatment with CAPE decreased ET-1 concentration and mediated a beneficial effect on parameters induced by sepsis.

Moreover, Ang II an octapeptide considered a powerful vasoconstrictor is produced by the angiotensin-converting enzyme (ACE) and plays a very important role in the regulation of cardiac functions [35,36]. In fact, elevated concentrations of Ang II have been found in the infarcted area, which have correlated with decreased conductance among myocytes [9]. Our results demonstrate that the infarction significantly increases the concentration of Ang II, while treatments with *Ro* and *Co* significantly decreased its concentration (Figure 4b). Using adult rats administered with rosmarinic acid, Liu et al. [37] found a decrease in the expression of ACE. Similarly, in our study, Ang II values decreased in *Ro*-treated rats. These results seem to be in agreement with the work of Orhan et al. [38], who found that treatment with *Co* inhibits ACE.

There is evidence that Ang II activates NOX4 via AT1 receptor pathway, which increases oxidative stress during ischemia [39]. We observed that the expression of NOX4 and p22phox subunits were increased in the left ventricle of infarcted animals; the treatment with both individual extracts and their combination reverted this effect (Figure 5a,b). This result is notably similar to that obtained in the study of Swaminathan et al. [23], where rats subjected to ischemia/reperfusion and administered with *Co* decreased NOX expression. Our study further demonstrated that Ang II production decreases with treatments; it is, therefore, reasonable to assume that a lower NOX4 activation exists. In consequence, the production of ROS by this pathway would decrease, thus favoring a higher antioxidant capacity (Figure 2a) that is accompanied by the decrease of the expression of three antioxidant enzymes (Figure 3).

### 3.3. Effect of Ro and Co, Compounds Administration on Vasodilators

Recent evidence shows that the vasopressor and proliferative actions of Ang II can be counteracted by Ang-(1–7) [11]. Loot et al. [40] demonstrated that Ang-(1–7) attenuates the process of cardiac failure that occurs after myocardial infarction. Furthermore, Liao et al. [41] demonstrated that Ang-(1–7) can inhibit ischemia reperfusion-induced oxidative stress and improve hemodynamic parameters in isolated rat hearts. In addition, Ang-(1–7) has been shown to decrease renal NOX levels and to attenuate NOX4 gene activation in kidneys of diabetic spontaneously hypertensive rats (SHR) [42]. Our results demonstrate that the concentration of Ang-(1–7) increases significantly with the administration of foliar extracts, but not with their combination (Figure 6a). This finding is in accordance with those published by other authors who used rosmarinic acid and observed an increase in ACE2 expression [37]. The increase in the concentration of Ang-(1–7) would lessen the concentration of ROS and might protect the heart during the period of ischemia. Furthermore, we observed that the administration of foliar ethanolic extracts favors the production of bradykinin (Figure 6b). Our results are consistent with other studies which described that both *Ro* and *Co* decreased the expression of ACE, which is one of the main bradykinin degrading enzymes [37,38].

Next, we evaluated eNOS protein expression. Our results show that during the ischemic process, the protein expression of eNOS decreases (Figure 7a). Previous reports have shown that ischemia activates cellular proteases [43]; therefore, it is likely that diminution of eNOS levels is due to protein degradation. Then, we evaluated the effect of foliar extracts and their combination on the concentration of biopterins. In ischemic rats, we found a decrease of BH_4_ concentration and, conversely, a concomitant rise in BH_2_—the oxidation product of BH_4_ (Figure 7b). Our results show that BH_4_:BH_2_ ratio diminished in rats subjected to MI and that treatment with the foliar extracts of *Ro* and *Co,* and their combination augmented the ratio (Figure 7c). The decrease in BH_4_:BH_2_ ratio has been correlated with low NO production in vitro and in vivo models [44]. Additionally, Vazquez-Vivar et al. [45] show that the decrease in BH_4_ concentration not only prevents the formation of NO by eNOS, but also augments the formation of O_2_^−^, hence becoming a potential radical source. It is known that NO regulates, among other processes, excitation–contraction coupling, heart rate, vegetative tone, and mitochondrial respiration. Alternatively, at the vascular level, NO plays a very important role in the control of angiogenesis and regulates tone, coronary perfusion, and capillary permeability [25,46,47,48,49].

### 3.4. Effect of Ro and Co Administration on Myocardial Structure and Function

An experimental study by Kaul [50] has reported that an increase in oxidative stress is accompanied by damage to heart structure and function. Some authors have shown that increasing ROS concentration decreases the activity of the Ca^2+^-dependent ATPase of the sarcoplasmic reticulum (SERCA) which, in turn, leads to a rise of approximately 50% in Ca^2+^ concentration [51]. This increase could also activate calpains which would then degrade key proteins in the cardiac structure [52].

Hertelendi et al. [53] have suggested that ROS oxidize the disulfide bonds present in the myofibrils causing myocardial stiffness and decreasing contraction. ROS can indirectly modulate the function of myofilaments through key protein kinases that induce post-translational changes of various proteins, such as troponin and myosin [54].

After the period of ischemia, the myocardium undergoes hypercontracture and damage to the cardiac fibers; mitochondria are also edematized, while in the nucleus, chromatin condensation is induced (Figure 8b,g,l). Our results indicate that treatment with *Ro*, *Co*, and their combination improves cardiac ultrastructure. Specifically, we observed that in rats treated with polyphenols contained in the extract, the structure of the cardiac fiber was very similar to that observed in the sham rats where bands I and A are clearly visible, and mitochondria also have a homogeneous structure. In addition, mild condensation of the chromatin was observed (Figure 8) compared to the severe condensation observed in MI-V. This change in nuclei morphology coincides with the concentration of 8-OH-2′dG (Figure 2c), where the infarction is accompanied by an increase in this DNA damage marker. Importantly, the administration of the foliar ethanol extracts decreases this parameter.

In this work, we demonstrate that natural compounds contained in *Ro* and *Co* improve cardiac function. Table 2 shows that the administration of polyphenols improves some of the cardiac parameters, such as left ventricular pressure (LVP) and CMW. These parameters are associated with diastolic function. However, in our assays we were unable to detect the effect of the extracts on in vivo cardiac function post MI. Other authors have already reported that antioxidants are beneficial after injury to the myocardium since they increase the force of myocardial contraction, increase coronary flow, limit the size of infarction, and prevent mortality. This protective effect is associated with the decrease in oxygen free radicals that can activate the production of inflammatory cytokines and the release of human neutrophil elastase from neutrophils [55,56]. It would be desirable to determine whether treatment with polyphenols could modify other processes such as: cellular K^+^ concentration, the duration of the potential, development of systolic strength, levels of high energy phosphate, and damage to the cellular metabolic machinery [57].

## 4. Materials and Methods

### 4.1. Preparation of Extracts of Crataegus oxyacantha and Rosmarinus officinalis

*Co* and *Ro* dried leaves were purchased from Future Foods^®^ and Comercializadora JR^®^, respectively. The extract was prepared as follows: 1 kg of dried leaves were powdered and placed in 6 L of methanol at 60 °C for 2 h. Then, it was filtred and the solution was concentrated to a final volume of 600 mL on a vacuum rotatory evaporator (Yamato Scientific Co., Ltd., Tokyo, Japan) [58]. The filtrate was air-dried and suspended in distilled water (200 mg/mL) to be administered to the animals. The analysis of the extracts was carried out using HPLC and spectrophotometric determination in the range of 700–200 nm [24,58].

### 4.2. High-Performance Liquid Chromatography (HPLC) Analysis

In order to identify polyphenolic constituents of *Ro* and *Co* extracts, we analyzed them by HPLC following previously reported methods [59,60]. Briefly, 20 µL of *Ro* extract was injected onto a reverse phase Hypersil H5 ODS column (50 mm × 4.6 mm i.d. × 3 μm) in a Waters Acquity H Class UPLC system (Milford, MA, USA), equipped with a UV-VIS detection system by diode arrangement. Separation and quantification were achieved at 25 °C using isocratic conditions with 0.1% phosphoric acid-60% acetonitrile as mobile phase, at a flow rate of 1.5 mL/min for 10 min. *Co* extract was analyzed injecting 20 μL of the extract onto a Nova-Pak C18 column (100 mm × 8 mm i.d. × 4 μm) Waters Acquity H Class UPLC system (Milford, MA, USA) equipped with a UV-VIS detection system by diode arrangement. The sample was eluted at a flow rate of 1 mL/min at room temperature, with a gradient mobile phase consisting of: solvent A (25 mM NaH_2_PO_4_ buffer containing 5% acetonitrile, pH 2.4) and B (25 mM NaH_2_PO_4_ buffer containing 25% acetonitrile, pH 2.4). The ratio of gradient A-B varied from 9:1 to 2:8 for the first 20 min, then maintained 35 min at 2:8 and changed back to 9:1 for further 5 min. The elution peaks were monitored at 360 nm. The identification of the compounds was based on the comparison of the actual retention time of the samples with their respective standards. 

### 4.3. Animals

Male Wistar rats (300 g) were bred and raised at our facilities and were separated in four different groups (*n* = 6 per group) to receive one of the sub-chronic (seven days) intraperitoneal (i.p) treatments: (a) vehicle in which the natural compounds were dissolved (2:1, water/ethanol), (b) *Rosmarinus officinalis* (*Ro*) extract (100 mg/kg/day), (c) *Crataegus oxyacantha* (*Co*) extract (100 mg/kg/day), and (d) *Ro+Co* combination (25 mg of Ro and 25 mg of Co/kg/day).

The Ethical Committee of the Instituto Nacional de Cardiología approved the study (Ministry of agriculture, SAGARPA, NOM-062-ZOO-1999, Mexico City, Mexico). The animals were maintained at standard conditions of light and temperature with water and food (LabDiet 5001; Richmond, IN, USA) ad libitum.

### 4.4. Myocardial Infarction (MI)

At the end of the treatment, anesthesia was induced (ketamine hydrochloride 80 mg/kg, and xylazine hydrochloride 10 mg/kg, i.m.) and subjected to myocardial infarction (MI). MI was induced by left anterior descending coronary artery (LAD) ligation with 7-0 PROLENE^®^ polypropylene suture. After 120 min of acute MI, the heart was cut out and the ischemic area separated to perform the analysis. A sham-operated group served as control [36,61].

### 4.5. Isolated Heart Perfused by the Langendorff Method

Left ventricular functional examination was performed in the heart of rats specifically assigned to this experimental phase (*n* = 6 per group) following a previously reported protocol [36]. The hearts were mounted into the Langendorff system and were perfused retrogradely via the aorta using a Grass stimulator (SIU5, Grass Instruments Co., Quincy, MA, USA). Heart rate (HR) was maintained at 312–324 beats per minute. Left ventricular pressure (LVP) was recorded using a Grass 79D (Grass Instruments Co., Quincy, MA, USA); coronary perfusion pressure (CPP) was also recorded by a pressure transducer (Gould P23ID, Gould Instruments, Cleveland, OH, USA). With the values of HR and LVP, CMW was calculated as HR × LVP = CMP. Coronary vascular resistance (CVR) was calculated using the following relationship: CPP/HR = CVR.

### 4.6. Structural Analysis by Electron Microscopy

For electron microscopy, ventricular tissue was fixed in 2.5% glutaraldehyde for 1 h, post-fixed in 1% osmium tetroxide, dehydrated with increasing concentrations of etanol and embeded in EPON 812 (Electron Microscopy Sciences, Hatfield, PA, USA). Ultrathin sections of 60 nm were cut using an Ultracut microtome (RMC pt XL, Boeckeler Instruments Inc., Tucson, AZ, USA); the sections were contrasted in uranyl acetate and lead citrate to be further examined with a JEM-1011 (JEOL Ltd., Tokyo, Japan) at 60 kV [36]. Random pictures of 2–3 myocardial ischemic areas were taken from three rats per group using 12,000× magnification for sarcomere and 20,000× magnification for mitochondria and nuclei.

### 4.7. Measurement of Total Antioxidant Capacity

TAC was determined following a previously reported method by Ibarra-Lara et al. [61].

### 4.8. Determination of Oxidative Stress Markers, and Vasoconstrictor and Vasorelaxant Agents

Ang II, Ang-(1–7), and MDA production was evaluated in myocardial ischemic areas from different experimental groups by capillary zone electrophoresis (CZE, P/ACE MDQ Capillary Electrophoresis System, Beckman Coulter, Inc., Fullerton, CA, USA) according to the methods described previously [36]. The production of BH_4_ and BH_2_ was determined by CZE as previously reported by Oidor-Chan et al. [44]. Data are expressed as pmol of BH_4_ or BH_2_ per mg of wet tissue. 

Endothelin-1 concentration was determined following the method reported by Kumarathasan et al. [62].

8-Hydroxy-2′-deoxyguanosine (8-HO-2dG) production was evaluated by the technique described by Kvasnicová et al. [63] and Tůma et al. [64]. Bradykinin was evaluated by capillary electrophoresis with a laser-induced fluorescence detector, as previously reported [65].

### 4.9. Western Blotting of SOD-Cu^2+^/Zn^2+^, SOD-Mn^2+^, Catalase, NOX4, p22phox and eNOS

Protein expression was analyzed in the myocardial ischemic area of rats from the different experimental groups. The frozen myocardial ischemic area was homogenized with a polytron (model PT-MR2100; Kinematica AG, Lucerne, Switzerland) (25% *w*/*v*) in a lysis buffer pH = 7.4 (250 mM Tris-HCl, 2.5 mM EDTA) and a mixture of protease inhibitors (10 μg/mL leupeptin and 20 μg/mL aprotinin) at 4 °C. Primary antibodies were from Santa Cruz Biotechnology (Santa Cruz, CA, USA) and secondary horseradish peroxidase-labeled antibodies were from Jackson Immunoresearch (Jackson Immunoresearch, Suffolk, UK). All blots were incubated with β-actin antibody as a control. Protein was detected with the Immobilon chemiluminescent system (Immobilon Western, Millipore, MA, USA) [35]. The immunoblotting signals were quantitated using a GS-800 densitometer (Bio-Rad Laboratories Inc., Hercules, CA, USA) and are reported as arbitrary units (AU).

### 4.10. Data Analysis

Results are expressed as the mean ± standard error of the mean (SEM). Experimental data were examined employing the one-way ANOVA followed by Tukey’s post hoc test. Differences are reported as statistically significant when *p* < 0.05.

## 5. Conclusions

Our results show that the treatment with extracts of *Rosmarinus officinalis* and *Crataegus oxyacantha* attenuate morphological and functional ischemic changes by both reducing oxidative stress and improving the balance between vasoconstrictors and vasodilators.

## Figures and Tables

**Table 1:** Migrations times of polyphenolic compound found in the extracts of *Rosmarinus officinalis* and *Crataegus oxyacantha*.

Extract	Compound	Migration Time (min)	Concentration ± SD (ng/mL)
*Medeeha Method 280 nm*			
*Rosmarinus officinalis*	Rosmarinic acid	6.112	5.214 ± 0.103
	Carnosol	8.193	4.758 ± 0.086
*Zhang Method 360 nm*			
*Crataegus oxyacantha*	Rutin	24.075	6.265 ± 0.093
	Quercetin	56.708	11.041 ± 0.118

**Table 2:** The effect of *Rosmarinus officinalis* (*Ro*), *Crataegus oxyacantha* (*Co*), and the combination of the extracts (*Ro+Co*) administration on hemodynamic parameters obtained by the Langendorff method.

	Sham	MI-V	MI-*Ro*	MI-*Co*	MI-*Ro+Co*
Left ventricular pressure (mmHg)	74.0 ± 1.3	56.8 ± 3.8*	69.2 ± 2.7	74.0 ± 1.3 Δ	70.0 ± 1.6
Coronary perfusion pressure (mmHg)	66.6 ± 5.4	88.0 ± 4.6	70.2 ± 4.6	73.8 ± 5.6	76.0 ± 8.7
Cardiac mechanical work (beats/min × mmHg)	24,808.0 ± 482.4	19,085.0 ± 1281.2 *	23,251.0 ± 922.6	24,864.0 ± 438.1 Δ	23,520.0 ± 541.8
Coronary vascular resistance (mmHg/mL/min)	6.7 ± 0.5	8.8 ± 0.5	7.0 ± 0.5	7.4 ± 0.6	7.6 ± 0.9

The values represent the mean ± SEM (*n* = 5 animals per group). Myocardial infarction (MI)-vehicle (MI-V) treated, MI-*Ro*, MI-*Co*, and MI-*Ro+Co*. * *p* < 0.05 vs. Sham, Δ *p* < 0.05 vs. MI-V. ANOVA one way post Tukey.
